# Targeting T cell exhaustion: emerging strategies in non-small cell lung cancer

**DOI:** 10.3389/fimmu.2024.1507501

**Published:** 2024-12-12

**Authors:** Xianqiang Liu, Xiaowei Xi, Shengshan Xu, Hongyu Chu, Penghui Hu, Dong Li, Bin Zhang, Hejie Liu, Tianxiao Jiang, Zhuming Lu

**Affiliations:** ^1^ Department of Thoracic Surgery, Jiangmen Central Hospital, Jiangmen, Guangdong, China; ^2^ Graduate School, Medical School of Chinese PLA, Beijing, China; ^3^ Technical University of Munich (TUM) School of Medicine and Health, Munich, Germany; ^4^ Department of Gastrointestinal, Colorectal and Anal Surgery, China-Japan Union Hospital of Jilin University, Changchun, China; ^5^ Scientific Research and Education Department, Jiangmen Central Hospital, Jiangmen, Guangdong, China; ^6^ Department of Intensive Care Unit and Clinical Experimental Center, Jiangmen Central Hospital, Jiangmen, China; ^7^ Department of Cardiovascular Disease and Clinical Experimental Center, Jiangmen Central Hospital, Jiangmen, China; ^8^ Department of Cardiology, The First Affiliated Hospital, Sun Yat-sen University, Guangzhou, China; ^9^ Department of General, Visceral, and Transplant Surgery, Ludwig-Maximilians-University Munich, Munich, Germany

**Keywords:** T cell exhaustion, immunotherapy, non-small cell lung cancer, cytokines, immune checkpoint inhibitors

## Abstract

Lung cancer continues to be a major contributor to cancer-related deaths globally. Recent advances in immunotherapy have introduced promising treatments targeting T cell functionality. Central to the efficacy of these therapies is the role of T cells, which are often rendered dysfunctional due to continuous antigenic stimulation in the tumor microenvironment–a condition referred to as T cell exhaustion. This review addresses the critical challenge of T cell exhaustion in non-small cell lung cancer (NSCLC), offering a detailed examination of its molecular underpinnings and the resultant therapeutic ineffectiveness. We synthesize current knowledge on the drivers of T cell exhaustion, evaluate emerging strategies for its reversal, and explore the potential impact of these insights for enhancing the clinical efficacy of immunotherapies. By consolidating reported clinical trials and preclinical studies, this article highlights innovative approaches to modulate immune responses and improve patient outcomes, thus providing a roadmap for future research and therapeutic development in lung cancer immunotherapy.

## 1 Introduction

Lung cancer continues to pose a significant global health issue, consistently ranking as the second most diagnosed malignancy as of 2020 and the leading contributor to cancer-related mortality. This disease is primarily divided into two subtypes: small cell lung cancer (SCLC) and non-small cell lung cancer (NSCLC) (1). SCLC, representing about 15% of all lung cancer instances, characterized by high aggressiveness and low survival rates, with a five-year survival rate of 7% (2). NSCLC comprises about 85% of cases and has a somewhat higher five-year survival rate of around 25% (3). Despite advancements in treatment, about half of all lung cancer patients are diagnosed with advanced stages of the disease, undergoing treatment regimens predominantly based on platinum-based chemotherapy (4). Even with the integration of chemotherapy and targeted therapies or other treatment modalities, the average five-year survival rate languishes at approximately 6%, underscoring a dire need for more effective therapeutic options (5).

In recent years, the emergence of immunotherapy has diversified the treatment modalities for lung cancer. T cells are vital for the effectiveness of immunotherapies, but their function can be significantly hindered by continuous antigen exposure from chronic infections or tumors, leading to T cell exhaustion. This state is marked by diminished production of effector cytokines such as IL-2 and IFN-γ, reduced proliferative capacity, and compromised cytotoxic and pro-inflammatory functions (6–8). The exacerbated expression of multiple immune checkpoints like PD-1, CTLA-4, LAG-3, TIGIT, BTLA, and TIM-3 further illustrates the impaired functionality of T cells under such conditions (9, 10). Consequently, many patients receiving immunotherapies like PD-1 or PD-L1 inhibitors do not maintain strong anti-tumor effects (11), propelling ongoing research to focus on methods to reverse T cell exhaustion (12). This review seeks to delve into emerging strategies to rejuvenate T cell activity, potentially counteracting the negative impact of continuous antigenic stimulation and fostering a more effective pathway for lung cancer treatment.

## 2 Inhibitory receptors involved in T cell exhaustion in lung cancer

Elevated expression of multiple inhibitory receptors has been well-documented in lung cancer patients, signifying a state of T cell exhaustion (13). Further complicating the treatment landscape, resistance to PD-1 inhibitors in lung cancer has been correlated with upregulation of other immune checkpoints such as TIM-3 and LAG-3 in mouse models (14).

### 2.1 PD-1

Programmed death receptor-1 (PD-1), a member of the CD28 transmembrane protein receptor family, plays a critical role in the immune system’s ability to respond to tumor cells. PD-1 suppresses the anti-cancer immune activity of T cells through its interaction with ligands PD-L1 or PD-L2 (15, 16), leading to downregulation of the T cell receptor and subsequent inhibition of T cell activation and cytokine release (17, 18). Notably, interferon-gamma activates the PI3K/AKT and JAK/STAT3 pathways in NSCLC, which enhances PD-L1 expression, a mechanism indicating IFN-γ induced immune suppression (19). Innovative treatments, such as vaccines using plasmacytoid dendritic cells in conjunction with anti-PD-1 therapies, have proved promise in amplifying the tumor-specific CD8+ T cell response in lung cancer (20). Other studies indicate that the miR-3127-5p/p-STAT3 axis (21), the EZH2-HIF-1α axis (22), KLF12 (23), and POU2F1 (24) are also involved in controlling PD-L1 expression, ultimately affecting T cell exhaustion. Therefore, a deeper understanding of the mechanisms that regulate PD-L1 expression could lead to improved strategies for enhancing the effectiveness of PD-L1/PD-1 inhibitors in lung cancer treatment. For a detailed summary of clinical trials employing PD-1 inhibitors in lung cancer, refer to **Supplementary Table 1**, which catalogues and summarizes these trials.

### 2.2 CTLA-4

As another inhibitory member of the CD28 family, cytotoxic T-lymphocyte-associated protein 4 (CTLA-4), mainly found on T cells, CTLA-4 competitively binds with the ligands CD80/CD86, curtailing CD28 signaling and subsequently reducing IL-2 production and T cell-APC contact time-crucially weakening T cell responses (25). This receptor also triggers the PI3-K pathway, diminishing T cell effectiveness further. The enhancement of PD-1/PD-L1 and CTLA-4 expression via the EGFR pathway suggests a potent avenue for immune suppression in lung cancer, exacerbated by ERK and NF-κB pathways (26). In a Phase Ib/II study (NCT04646330), researchers assessed the combined use of cadonilimab (a PD-1/CTLA-4 bispecific antibody) with anlotinib as a primary treatment for advanced NSCLC, revealing the regimen’s safety and potential efficacy at 10 mg/kg every three weeks. Employing anti-PD-1/PD-L1 and anti-CTLA-4 drugs targets different stages of the cancer immunity cycle; their combined use can produce a synergistic effect, helping to overcome resistance to monotherapy (27, 28). Additionally, **Supplementary Table 2** provides an overview of clinical trials investigating CTLA-4 inhibitors in lung cancer.

### 2.3 TIM-3

T cell immunoglobulin and mucin-domain containing-3 (TIM-3), a member of the immunoglobulin superfamily, was first identified on CD4+ Th1 and CD8+ Tc1 cells as a surface marker that binds to galectin-9. This interaction negatively regulates the function of these cells (29, 30). TIM-3 is also found on a variety of cells within the tumor microenvironment (TME), such as NK cells, dendritic cells (DCs), and tumor cells, which underscores its pivotal role in disease progression in lung cancer (30, 31). High levels of TIM-3 on NK cells and tumor-associated macrophages are related to advanced disease stages and reduced survival rates in lung cancer patients (32, 33). Therapeutically, combining anti-TIM-3 with anti-PD-1 therapies has been effective in reviving the effector functions of CD8 T cells *in vitro*, indicating that TIM-3 plays a significant role not only as a biomarker of resistance but also in the mechanisms of resistance itself. A Phase I/II trial (NCT02608268) evaluating MGB453 (an anti-TIM-3 drug) combined with PDR001 (an anti-PD-1 therapy) demonstrated good safety and antitumor activity, highlighting the clinical potential of targeting TIM-3 in advanced NSCLC (34). Ongoing clinical trials continue to assess the efficacy of several novel TIM-3 inhibitors, including AZD7789, INCAGN02390, BMS-986258, cobolimab, and sabatolimab (MBG453) (35).

### 2.4 LAG3

Lymphocyte activation gene-3 (LAG-3) exhibits structural similarities to CD4 in its extracellular domain, yet its intracellular domain markedly differs, indicating unique functional attributes (36). LAG-3 more strongly binds MHC-II compared to CD4, effectively interfering with CD4’s interaction with MHC-II, ultimately suppressing T cell activation (37). Unlike naïve T cells, LAG-3 is expressed on CD4+ and CD8+ T cells after antigen exposure, serving to inhibit their proliferation (38, 39). In the TME, constant antigen exposure heightens LAG-3 expression, contributing to T cell exhaustion and impaired tumor cell elimination. T cells that express both LAG-3 and PD-1 exhibit a greater degree of exhaustion than those expressing PD-1 alone, making LAG-3 a potential target for rescuing dysfunctional T cells in lung cancer. A Phase II study (NCT03365791) evaluating LAG525 combined with PDR001 in advanced solid tumors showed promising anti-tumor effects, achieving a 24-week clinical benefit rate of 27% in SCLC cases. Additionally, Xencor’s bispecific antibody XmAb^®^22841 targets both CTLA-4 and LAG-3, boosting T cell activation and proliferation (40). A Phase I study (NCT03849469) is currently exploring the optimal dose of XmAb22841, alone or with pembrolizumab, for treating advanced solid tumors, including SCLC (41).

### 2.5 TIGIT

The T cell immunoreceptor with Ig and ITIM domains (TIGIT) is a recently identified immune checkpoint receptor primarily found on T cells and NK cells, binding to the ligand CD155. TIGIT serves as a marker for T cell exhaustion, effectively identifying exhausted T cells across different stages of differentiation and fostering the exhaustion of CD8 T cells and NK cells under chronic conditions (42–44). Furthermore, research by Yang et al. found that the positivity rate of CD155 in the immunohistochemistry results of squamous lung cancer is significantly higher than that of PD-L1, indicating a crucial role for the TIGIT/CD155 axis in the development and progression of this cancer type (45). Additionally, high TIGIT levels are linked to greater severity in lung adenocarcinoma, and patients with overexpressed CD155 in lung adenocarcinoma and SCLC have shorter progression-free survival and overall survival (46–48). Studies show that antagonistic antibodies against TIGIT, when used with PD-1 inhibitors, effectively curb lung cancer growth in immunocompetent mice (49). Ongoing clinical trials, such as NCT02964013 (MK-7684-001), are assessing the efficacy of the anti-TIGIT antibody vibostolimab in combination with pembrolizumab, demonstrating promising antitumor effects particularly in patients new to anti-PD-1/PD-L1 therapy and those with PD-L1 positivity (50).

### 2.6 BTLA

The B and T lymphocyte attenuator (BTLA) belongs to the immunoglobulin superfamily and is primarily found on the surfaces of T cells, B cells, NK cells, and DCs (51, 52). As an inhibitory receptor, BTLA limits the activation, proliferation, and production of pro-inflammatory cytokines (IFN-γ, IL-2, and IL-10) in T and B cells through its interaction with the ligand HVEM (53). In lung cancer, BTLA plays a particularly important role, co-participating in T cell exhaustion with other inhibitory receptors like PD-1 and CTLA-4 (54, 55). Within the TME, elevated BTLA levels result in reduced T cell efficacy, affecting their immune response to tumors (56). Studies by Mittal et al. and Thommen et al. demonstrate that BTLA, along with markers like PD-1 and TIM-3, is upregulated in T cells post-tumor implantation in mice and in infiltrating CD8 T cells in advanced lung cancer patients, underscoring its role in T cell dysfunction during tumor progression (57, 58). Clinical studies suggest that high expression of BTLA is linked to unfavorable outcomes in NSCLC and is viewed as a new target for immunotherapy, potentially by combining other therapeutic strategies (such as PD-1 blockade) to restore T cell function (59).

### 2.7 IDO

Indoleamine 2,3-dioxygenase (IDO) is an intracellular enzyme that catalyzes the conversion of the essential amino acid L-tryptophan into N-formylkynurenine (60). By diminishing local tryptophan levels and augmenting the production of immunoregulatory metabolites, IDO exhibits its immunosuppressive effects (61). These metabolites not only inhibit T lymphocyte proliferation but also promote apoptosis and drive the transformation of naïve T cells into regulatory T cells. Additionally, overexpression of IDO in DCs affects their maturation, reducing antigen presentation and decreasing co-stimulatory molecule expression (60). Increasing evidence links IDO overexpression with adverse outcomes in several cancers (62, 63). IDO is considered a mechanism of resistance that might hinder the effectiveness of checkpoint inhibitor therapies. In a mouse model of lung cancer, silencing IDO1 inhibited tumor growth by reversing T cell exhaustion. Furthermore, blocking the T cell exhaustion induced by IDO1 can enhance the performance of PD-1 inhibitors in lung cancer treatment (64). Therefore, IDO as a potential immunosuppressant is worth further exploration. **Table 1** provides a comprehensive overview of the clinical trials involving immune checkpoint modulators, such as TIM-3, LAG-3, TIGIT, BTLA, and IDO, in the context of lung cancer.

**Table 1:** Clinical trials of immune checkpoint modulators (TIM3, LAG-3, TIGIT, BTLA, and IDO) in lung cancer.

Drug	Target	Clinical trial ID	Phase
TIM3			
AZD7789	TIM-3/PD-1	NCT04931654	I/II
Lomvastomig	TIM-3/PD-1	NCT03708328	I
TSR-022	TIM-3	NCT06322693	I
INCAGN02390	TIM-3	NCT03652077	I
S095018 plus Cemiplimab	TIM-3	NCT06162572	I/II
LAG-3			
TSR-033	LAG-3	NCT03250832	I
INCAGN02385	LAG-3	NCT03538028	I
MGD013	LAG-3	NCT03219268	I
PDR001+LAG525	LAG-3	NCT03365791	II
RO7247669	LAG3/PD-1	NCT04140500	I/II
XmAb®22841	LAG-3/CTLA-4	NCT03849469	I
Fianlimab plus Cemiplimab	LAG-3	NCT05800015/NCT05785767	II/III
HLX26 plus Serplulimab	LAG-3	NCT05787613	II
Eftilagimod α plus Pembrolizumab	LAG-3	NCT03625323	II
TIGIT			
AZD2936	TIGIT/PD-1	NCT04995523	I/II
HLX301	TIGIT/PD-1	NCT05102214	I/II
EOS-448	TIGIT	NCT05060432	I/II
COM902	TIGIT	NCT04354246	I
Ociperlimab	TIGIT	NCT04952597	II
Domvanalimab	TIGIT	NCT04736173	III
Domvanalimab plus Zimberelimab	TIGIT	NCT04262856/NCT04736173 /NCT04791839	II/III
Ociperlimab plus Tislelizumab	TIGIT	NCT04746924/NCT05014815	II/III
IBI939 plus Sintilimab	TIGIT	NCT04672356/NCT04672369	I
Tiragolumab plus Atezolizumab	TIGIT	NCT04294810	III
Tiragolumab plus Atezolizumab	TIGIT	NCT03563716	II
BTLA			
TAB004	BTLA	NCT04137900	I
HFB200603	BTLA	NCT05789069	I
Tifcemalimab	BTLA	NCT06095583	III
JS004 plus Toripalimab	BTLA	NCT05664971/NCT06256237/ NCT05000684/NCT05891080	I/II
IDO			
IDO peptide vaccination	IDO	NCT01219348	I
BMS-986205 plus nivolumab	IDO	NCT02658890	I/II
Epacadostat plus Pembrolizumab	IDO	NCT03322540	II
Pembrolizumab plus Epacadostat	IDO	NCT03322566	II
IO102-IO103 plus Pembrolizumab	IDO	NCT05077709	II

## 3 The impact of cytokines on T cell exhaustion in lung cancer

### 3.1 IL-10

IL-10, a key anti-inflammatory cytokine, is secreted by various immune cells, such as DCs, B cells, CD8 T cells, and non-regulatory CD4 T cells. It plays a critical role in maintaining self-tolerance and protecting against tissue damage in inflamed conditions by inhibiting Th17 cell-mediated inflammation and reducing levels of TNF-α and IL-6 (65, 66). IL-10 exhibits a complex, dual role in antitumor immunity, with its effects shaped by various factors, including cancer type, cell type, TME conditions, and IL-10 concentration. In models of breast cancer, cutaneous squamous cell carcinoma, and lymphoma, IL-10 deficiency correlates with increased tumor burden and impaired immune surveillance, along with elevated rates of CD8+ T cell exhaustion (67, 68). Conversely, in colorectal cancer, bladder cancer, and melanoma models, IL-10 has been shown to promote CD8+ T cell exhaustion, thereby weakening antitumor immunity (69–72). Additionally, in non-small cell lung cancer, IL-10, in conjunction with Tregs and IL-35, is believed to further exacerbate T cell exhaustion, suppressing antitumor responses (70).

The concentration of IL-10 plays a critical role in determining its antitumor effects. High levels of IL-10 can stimulate CD8+ T cell proliferation and enhance cytotoxicity, while low levels tend to exert immunosuppressive effects, diminishing the antitumor immune response (68). In chronic inflammatory settings, IL-10’s anti-inflammatory properties may help maintain CD8+ T cell antitumor functions (73, 74). Furthermore, IL-10 influences CD8+ T cell functionality through metabolic reprogramming, particularly by inducing oxidative phosphorylation (OXPHOS) pathways (75, 76). This upregulation of OXPHOS can partially reinvigorate exhausted T cells, enhancing their proliferative capacity, effector functions, and overall antitumor immunity (76). IL-10’s effects also vary among different cell types. For example, in B cells, IL-10 promotes proliferation, stimulates immunoglobulin secretion and isotype switching, and enhances tumor-killing capabilities (77, 78). Future research should focus on the role of IL-10 in relation to exhausted CD8+ T cells in non-small cell lung cancer, as well as the key factors influencing this dynamic, to improve the efficacy of lung cancer immunotherapies.

### 3.2 IFNα/β

IFNα/β are pro-inflammatory cytokines with various anti-tumor activities, such as direct tumor cell eradication and the stimulation of immune cells like DCs and CD8 T cells (79–81). Studies suggest that IFNα/β can trigger the expression of IL-10, PD-L1, and other inhibitory regulators, potentially leading to T cell exhaustion through the influence on the transcription factor Tcf1 (82, 83). To date, IFNα/β has been approved for treating multiple malignancies, such as renal cell carcinoma and melanoma. Overall, the efficacy of combining PD-1/PD-L1 inhibitors with IFNα/β therapy is influenced by various factors, including the type and dosage of IFNα/β, timing and duration of treatment, patient immune status, and cancer type. Thus, understanding the mechanisms of action of IFNα/β and its interaction with PD-1/PD-L1 combined therapy is crucial for optimizing cancer treatment strategies.

### 3.3 TGF-β

TGF-β, a versatile cytokine, plays a critical role in regulating cell growth and differentiation. It also fosters T cell exhaustion; notably, TGF-β’s stimulation of exhausted T cell precursors leads to the inhibition of mTOR signaling, which in turn produces inhibitory cytokines that dampen the immune response (84). Furthermore, TGF-β reduces or suppresses immune cell activation through triggering SMAD transcription regulators downstream. Tumor cells release TGF-β, which directly increases PD-1 transcription in these cells, contributing to T cell exhaustion. Consequently, blocking TGF-β signaling could directly bolster anti-tumor immunity (85). The significant link between TGF-β signaling and impaired immune responses in lung cancer underscores the need to focus on this pathway when developing new therapeutic strategies. Extensive research is required to unravel its full mechanisms and evaluate the effectiveness of TGF-β inhibitors in treating lung cancer.

### 3.4 IL-2

IL-2, commonly referred to as T cell growth factor, is integral to T cell proliferation. At initial tumor growth stages, IL-2-driven BLIMP1 expression is vital for the development and differentiation of CD8 T cells. Recent findings suggest that IL-2 signaling can alter the differentiation trajectory of exhausted CD8 T cell precursors, potentially reversing T cell exhaustion (86). However, studies indicate that IL-2 can enhance 5-HTP production through the STAT5-TPH1 pathway, which activates the aryl hydrocarbon receptor and may lead to CD8 T cell exhaustion (87). Thus, IL-2 serves a dual function: it both contributes to and could potentially reverse the exhausted T cell phenotype. Currently, combining IL-2 with PD-1 inhibitors has demonstrated promising synergistic effects in reactivating exhausted CD8 T cells (88, 89). Ongoing research is crucial to fully ascertain the therapeutic impact of IL-2 in lung cancer treatment.

## 4 Non-cytokine mediators of T cell exhaustion

Beyond cytokines, non-cytokine factors such as metabolites and metabolic fuel sources like adenosine, cholesterol, and fatty acids, also contribute to T cell exhaustion (90, 91).

### 4.1 Prostaglandin E2

The arachidonic acid pathway, involved in immune suppression across various cancer types, includes the enzyme microsomal prostaglandin E2 synthase-1, downstream of cyclooxygenase 2, which limits the body’s anti-tumor immunity. Prostaglandin E2 (PGE2), known for its immunosuppressive effects, can suppress the activation and proliferation of immune cells such as NK cells and B cells via the PGE2-EP signaling pathway (92–94). Recent research in lung cancer has shown that PGE2 promotes immune tolerance by upregulating PD-1 on infiltrating CD8 T cells, thereby contributing to their exhaustion (95). Consequently, targeting the PGE2-EP signaling pathway presents a promising approach to counteract T cell exhaustion in lung cancer therapy.

### 4.2 Adenosine

Adenosine (Ado) is produced from extracellular ATP through the actions of ectonucleotidases CD39 and CD73 on cell surfaces (96). Persistent high levels of Ado can contribute to an immunosuppressive microenvironment, although the precise mechanisms remain unclear (97). Ado impairs the activation of CD8 T cells in the TME and disrupts their tumor recognition capabilities, mainly through the A2AR/PKA/mTORC1 signaling pathway (98). Additionally, Ado further dampens tumor immunity by decreasing the infiltration of immune cells into tumors (99). Due to poor vascular development in tumors, the TME often exists in a hypoxic state, facilitate Ado production and activate immunosuppressive A2A and A2B receptors. Shifts in tumor metabolism towards glycolysis and the resulting increase in lactate also lower pH levels, promoting M2 polarization and inhibiting T cell activation (100, 101). Additionally, studies by Maj and colleagues have shown that during checkpoint therapy, the apoptosis of cancer cells and Tregs leads to an increase in ATP release, which is subsequently transformed into adenosine by the enzymes CD39 and CD73. The increased Ado in the TME counteracts the effects of checkpoint therapy, further suppressing anti-tumor immune responses (102). This suggests that Ado may be a potential target for advanced immunotherapies. Currently, a phase I clinical trial involving CPI-444 (an A2AR antagonist) combined with the PD-1 inhibitor nivolumab for treating non-small cell lung cancer is underway (NCT0265582).

### 4.3 Cholesterol

Cholesterol, a vital component of cell membranes, affects membrane fluidity as well as gene expression and metabolism, and impacting anti-tumor immunity (103). Its role in T cell activation is debated; some studies indicate it may suppress TCR signaling either by binding to the transmembrane region of TCRβ or by interfering with TCR oligomerization (104). In the TME, cholesterol is known to promote the expression of immunosuppressive receptors, leading to CD8 T cell exhaustion. Additionally, research has found that cholesterol levels in tumor-infiltrating CD8 T cells correlate with exhaustion status and the presence of immune checkpoints such as PD-1, TIM-3, and LAG-3 (104, 105). Statins, the primary compounds inhibiting cholesterol synthesis, are highly safe, with atorvastatin reported to downregulate the expression of inhibitory receptors like LAG-3, PD-1, TIM-3, and CTLA-4 in T cells, indirectly indicating the relationship between cholesterol and immune checkpoint expression (106). Moreover, statins have been found to reduce cancer-related mortality by 15% (107). Therefore, cholesterol-lowering medications may be combined with immunotherapy for cancer patients, offering new possibilities for treatment.

### 4.4 Clinical implications of T cell exhaustion in lung cancer immunotherapy

**Figure 1:**
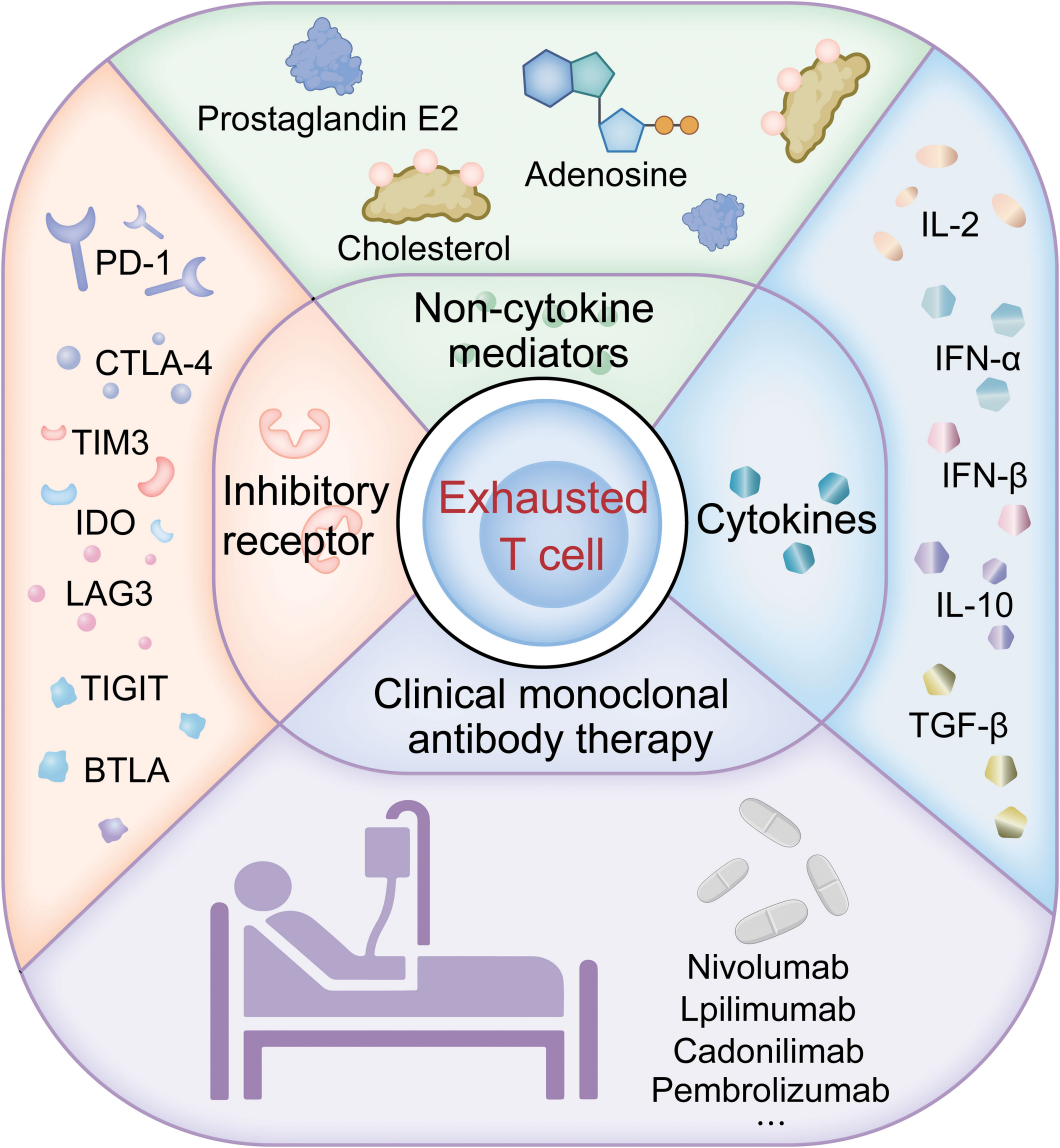
Pathways of T cell exhaustion and therapeutic interventions in lung cancer.

Understanding T cell exhaustion is crucial for monitoring and treating lung cancer. **Figure 1** provides an overview of the pathways of T cell exhaustion and therapeutic interventions in lung cancer currently under study. Exhausted T cells are characterized by the expression of inhibitory receptors such as PD-1 and TIM-3, with their co-expression often signaling severe exhaustion and correlating with a poorer prognosis in lung cancer. In the TME, the presence of these markers on CD8 T cells can predict the response to immune checkpoint inhibitor therapy. Utilizing transcriptomics and multiplex staining techniques to identify patients who may benefit from ICI therapy is increasingly valuable in clinical research. Approaches to reverse T cell exhaustion and enhance anti-tumor immunity are promising, yet obstacles like immunosuppressive cytokines, certain immune cells, and elevated inhibitory receptors in the TME all play roles in promoting T cell exhaustion, which in turn dampens anti-tumor responses. Studies show that blocking inhibitory receptors and cytokines can effectively reverse T cell exhaustion in cancer patients, particularly in those with advanced disease, enhancing anti-tumor immunity. Clinical application of anti-PD-1 antibodies has been successful, and blocking PD-1 or PD-L1 improves the functionality of exhausted CD8 T cells (9, 108, 109), indicating that T cell exhaustion is not irreversible. Preclinical studies confirm that anti-PD-1 immune checkpoint inhibitors can effectively ameliorate T cell exhaustion and are more effective when combined with other immune checkpoint inhibitors (110); This has led to FDA approval for combination therapies like nivolumab plus ipilimumab (111). While single-agent CTLA-4 or PD-1 blockade has significantly extended survival in some cancer patients, most do not respond. Therefore, strategies against T cell exhaustion are becoming a focus for enhancing the effectiveness of anti-tumor therapies, especially in enhancing the efficacy and durability of treatments like CAR-T cell therapies.

## 5 Discussion and conclusion

Recent advances in immunotherapy have expanded treatment options for lung cancer, with T cell exhaustion emerging as a key area of interest in the disease’s pathogenesis, progression, and therapy. While the precise mechanisms behind T cell exhaustion continue to be studied, further research and clinical trials are essential to establish clear findings. Additionally, the exploration of T cell dysfunction presents a new avenue in immunotherapy. A thorough understanding of T cell exhaustion and its underlying mechanisms is critical to comprehending the immune dynamics in lung cancer. This knowledge is fundamental to crafting innovative treatment approaches for this challenging disease.
